# “Prevalence of orthorexia nervosa in a sample of patients attending sligo/leitrim mental health services with a diagnosis of eating disorder”

**DOI:** 10.1192/j.eurpsy.2021.940

**Published:** 2021-08-13

**Authors:** I. Graffeo, M. Harron, E. O’Mahony

**Affiliations:** 1 Sligo/leitrim Mental Health Services, St Columba’s Hospital, Sligo, Ireland; 2 Psychiatry, Sligo/Leitrim Mental Health Services, Sligo, Ireland

**Keywords:** eating disorders, orthorexia nervosa, anorexia nervosa, ocd

## Abstract

**Introduction:**

The term Orthorexia derives from the Greek “ortho – correct” and “orexis – appetite”; Orthorexia Nervosa is a pathological fixation with healthy eating that, starting with the idea to obtain a maximum health with a proper diet, leads to malnourishment and other medical sequelae, loss of relationships, loss of self-esteem, poor quality of life in general. Orthorexia, despite receiving broad empirical evidence, is not currently included in any psychiatric diagnostic manual.

**Objectives:**

The main aim of this study is to investigate its presence in a sample of patients already diagnosed with a canonical eating disorder and also to understand eventual overlaps with other clinical disorders in order to optimize treatment and follow up.

**Methods:**

The ORTO-15 questionnaire, developed by an Italian team of researchers in 2005, was used to achieve the above aims: it is a tool comprehensive of 15 questions that assesses eating habits perceived as healthy. Really interesting and fascinating is to comprehend if people with a diagnosis of eating disorder present orthorectic behaviour and how this emerging reality fits in the Irish society with its peculiarities and uniqueness.

**Results:**

The Point Prevalence obtained is 17.9%. The expected rates of Orthorexia Nervosa in the general population are between 6.9% and 57.6%, with a peak of 81.8% in specific populations, fact that places our examined sample in the lower side of the prevalence previously considered in other studies.

**Conclusions:**

It is significant the absence of correlation found between OCD and ON and that ON is more linked to Bulimia Nervosa rather than Anorexia Nervosa.

